# The Book World of Medicine and Science

**Published:** 1900-07-21

**Authors:** 


### The Book World of Medicine and Science.

Burdett's Hospitals and Charities, 1900: Being the Year Book of Philanthropy and the Hospital Annual. Containing a review of the position and requirements, and chapters on the management, revenue, and cost of the charities. By Sir Henry Burdett, K.C.B., (London: The Scientific Press, Ltd., 28 and 29, Southampton Street, Strand, W.C. New York: Charles C. Scribner's Sons. Price 5s.)

This useful publication is now in its eleventh year, and constitutes a fund of information regarding the progress of the hospital world which no one interested in medical philanthropy can afford to be without. It is, indeed, invidious to limit its usefulness to things medical, as there are chapters dealing with missions, orphanages and rescue homes, and institutions for the help of the blind, the deaf, and the dumb. The book is, however, above all things, the "Hospital Annual," and it contains a vast amount of information, not to be obtained elsewhere, concerning London, provincial, colonial, and American hospitals. We are inclined to think that some of the institutions given are not altogether worthy of their position in this volume. Thus, some hospitals have not responded to the request for details of their constitution, accommodation, &c., for several years. Might not such institutions be omitted? It seems a pity to occupy valuable space with information which may no longer be accurate. In the prefatory chapters of the annual, which are always full of interest, will be found this year some very interesting statistics as to the financial position of the London hospitals. These show that this is not by any means so bad as popular sentiment, aided, perhaps, by doleful advertisements, generally believes. Not that these advertisements are either false or unjustifiable. "Annual expenditure £20,000, annual income £10,000" is a perfectly true proportionate statement of the financial position of many hospitals; but it simply means that the invested funds of the hospital meet only half its average expenditure, and that the remainder must be made up by subscriptions. This is, in fact, the exact position of affairs. As Sir Henry Burdett shows, about 50 per cent. of the hospital income of London comes from invested funds; 13 7 per cent. from annual subscriptions; from donations, 15·8 per cent.; Prince of Wales's Fund, 7·9 per cent.; Hospital Sunday Fund, 4 5 per cent.; Hospital Saturday Fund, 1·5 per cent.; independent contributions from workpeople, 0·8 per cent.; nurses' fees and profit of nursing institutions, 1·7 per cent.; and miscellaneous receipts, 0·5 per cent. This list shows, indeed, that the friends of hospitals must be unremitting in their endeavours to keep up the income to the level of the expenditure; but it does not show, as some people would fain make out, that the adequate support of our hospitals by means of voluntary effort is impossible. On the contrary, there is every encouragement for clinging to it. Many of the London hospitals have invested funds equal to from three to over five years' entire expenditure, and may therefore be regarded as being on a financially sound basis. That they should prefer to keep these funds intact and spend only the income of them is only common sense; and, as a matter of fact, they are, as Sir Henry Burdett shows, none the worse for having to ask the public annually for half of their income. As he says, "The history of our country and nation has shown over and over again that when once an institution possesses reserve funds to so large an amount that its managers are practically independent of public support they are apt to grow careless of public criticism." This admitted fact is the best justification of the voluntary system. It is, however, likely that more money could be got for our hospitals if a persistent attempt was made to reach those who can afford only a little. The contributions which are taken up in many workshops help to this end in one way, and the new League of Mercy, for which the Queen has granted a charter, and in which the Prince of Wales has taken a great interest, will give valuable assistance in another. Each member of the League of Mercy pledges himself or herself to raise—not give—£1 per annum for our hospitals. This may be done by obtaining twenty subscribers of a shilling or upwards, or by getting one honorary member who will subscribe a guinea. We advise members of the league to devote their attention to the donors of the modest shillings, for every £1 thus raised means that twenty more persons are taking a living interest in the work of our hospitals, and it is the living interest of the public, not less than the money that is given, that is the best guarantee of the prosperity and integrity of the voluntary system. The offices of the league are at 29, Southampton Street, Strand.

### NOTES ON BOOKS.

John Bull's Trip to Paris (The "Favourite" Magazine), although not a guide book, partakes much of the character of that sort of literature, being in fact a slight aud sketchy description of the Paris of to-day. It is brightened up and made interesting by a considerable number of illustrations, and will doubtless prove acceptable to many readers.

To-day is the day of shilling maps. The constant shifting of interest from one quarter of the globe to another, as war follows war, or discovery follows discovery, renders maps essential to the proper comprehension of current events, and there is this to be said for the map-maker that he is never many days behindhand in producing just what one requires. Of this the latest example is Bacon's new large print Map of China (G. W. Bacon and Co.), which has just been published. It teaches many things, and will be found most conducive to a clear understanding of what is going on in that part of the world at the present moment.

The British Architect for July 13th contains some good illustrations of the Bedford County Hospital, the opening of which last year we noticed at the time. They show more especially the faïence decorations in the children's ward (by Messrs. Simpson), which not only form an admirable washable wall surface, but, from their illustrating nursery rhymes and stories, are highly appropriate for their position.

